# Air Pollution and Parkinson’s Disease Pathology: Clinical Evidence and the Molecular Mechanisms Linking Airborne Toxicants to Neuroinflammation and Neurodegeneration

**DOI:** 10.1155/padi/6914112

**Published:** 2026-08-10

**Authors:** Sina Pakkhesal, Seyedyashar Pourebrahimian Leilabadi, Sina Hamzehzadeh, Farshad Zare, Elnaz Gholipour-Khalili, Fatemeh Malekinejad, Shahrzad Fakhkhari, Mahnaz Talebi, Amirreza Naseri

**Affiliations:** ^1^ Student Research Committee, Tabriz University of Medical Sciences, Tabriz, Iran, tbzmed.ac.ir; ^2^ Research Center for Evidence-Based Medicine, Iranian EBM Centre: A Joanna Briggs Institute (JBI) Center of Excellence, Tabriz University of Medical Sciences, Tabriz, Iran, tbzmed.ac.ir; ^3^ Medical Faculty, Yeditepe University, Istanbul, Türkiye, yeditepe.edu.tr; ^4^ Research Center for Integrative Medicine in Aging, Aging Research Institute, Tabriz University of Medical Sciences, Tabriz, Iran, tbzmed.ac.ir; ^5^ Neurosciences Research Center (NSRC), Tabriz University of Medical Sciences, Tabriz, Iran, tbzmed.ac.ir; ^6^ Tabriz USERN Office, Universal Scientific Education and Research Network (USERN), Tabriz, Iran, usern.tums.ac.ir

**Keywords:** air pollution, gut microbiota, inflammation, Parkinson’s disease, particulate matter

## Abstract

Parkinson’s disease (PD) is a neurodegenerative condition, and its rising prevalence necessitates the urgent need to identify modifiable risk factors. Air pollution, a pervasive and also escalating public health problem, has emerged as a potential contributor to the incidence and progression of PD. As urbanization and industrialization continue to exacerbate global air pollution levels, understanding the relationship between airborne toxicants and PD is a pressing scientific and public health priority. In this review, we critically discuss the current epidemiological evidence and the cellular and molecular mechanisms underlying air pollution‐induced neurodegeneration in PD. Clinical studies associate long‐term interaction with pollutants such as nitrogen oxides, particulate matter, and ozone with increased PD risk. Evidence also suggests that air pollution worsens PD prognosis. Mechanistically, air pollution is hypothesized to contribute to PD pathogenesis through gut microbiome alternations, oxidative stress, and neuroinflammatory pathways, as well as promoting α‐synuclein aggregation.

## 1. Introduction

Parkinson’s disease (PD) is a widespread neurodegenerative condition correlated to aging, manifested by signs and symptoms including tremors, stiffness of muscles, inflexibility, and slowed movements [1]. The occurrence of PD escalates by advancing age, affecting around 1% of those aged 60 and above. Increasing trends in the burden of PD from 1990 to 2019 have been observed in most regions, including Eastern Europe and high‐income North America [2], which makes it one of the neurological conditions with the greatest rate of growth. Estimates for PD cases in the year 2040 vary between 12 and 17 million [3]. Only in the United States in 2017, PD imposed $25.4 billion of direct medical expenditures along with $26.5 billion indirect and also nonmedical charges, which made an overall economic burden of about $51.9 billion [4]. According to a meta‐analysis in 2024, the cost of disease for PD averages €20,911.37 per patient annually, with healthcare costs accounting for 46.1%, productivity loss for 37.4%, and costs to patients and families for 16.4% [5]. PD is characterized by the gradual loss of dopaminergic neurons located in the substantia nigra, accumulation of uncommon proteins such as α‐synuclein, and neuron loss in specific brain regions [6], leading to motor dysfunction, cognitive impairment, and various nonmotor symptoms. While the underlying pathogenesis of PD is multifactorial, research indicates that environmental factors, particularly air pollution, may be crucial in the onset and progression of the disease [7–9].

According to the World Health Organization (WHO)’s definition, air pollution is known as the existence of one or more pollutants in the environment, such as gas, dust, fumes, odor, mist, smoke, or vapor, in amounts and duration that may be harmful to health, which can lead to oxidative stress, inflammation, immune system suppression, and the ability of cells to mutate [10], and it can be either indoor or outdoor. Air pollution is an established risk factor for lung cancer [11], chronic obstructive pulmonary disease (COPD) [12], ischemic heart disease [13], and stroke [14]. Contaminants in the atmosphere usually include particulate matter (PM), carbon monoxide (CO), nitrogen oxide (NO_x_), ozone (O3), benzene, polycyclic aromatic hydrocarbons (notably benzo‐a‐pyrene‐BaP), sulfur oxides (SO_x_) and various metals like manganese (Mn), arsenic (As), cadmium (Cd), lead (Pb), mercury (Hg), nickel (Ni), aluminum (Al), and vanadium (V) produced by industrial emissions, vehicular traffic, and biomass burning. WHO established air quality guideline levels in 2005, setting the permissible concentration of PM2.5 at 25 μg/m^3^ for a 24‐h exposure and a yearly average of 10 μg/m^3^. For PM10, the daily exposure limit was set at 50 μg/m^3^ with an annual average number of 20 μg/m^3^. However, the guidelines were revised in 2021, reducing the 24‐h exposure limit for PM2.5–15 μg/m^3^ and the average for every year to 5 μg/m^3^, while the daily exposure limit for PM10 was adjusted to 45 μg/m^3^ with a 15 μg/m^3^ average for a year. Regarding O3, this level was set at 100 μg/m^3^ for a 24‐h exposure [15, 16].

By synthesizing the current evidence from epidemiological, experimental, and mechanistic studies, the intention of this narrative review is to present a comprehensive overview of the potential correlation between PD and air pollution.

## 2. Method

A search was conducted in PubMed from database inception to February 2026. The search strategy combined terms for air pollution (e.g., “air pollutant,” “particulate matter,” “PM2.5,” “PM10,” “nitrogen oxides,” “ozone,” “traffic‐related emission,” and “metal exposure”) with “Parkinson disease.” Original research articles, meta‐analyses, systematic reviews, and narrative reviews published in English were considered. The present narrative synthesis prioritizes epidemiological and experimental studies that attend to the association between air pollution and PD.

## 3. Air Pollution, PD Incidence, and Possible Mediators

Studies have assessed the influence of atmospheric particulates on PD development (Table 1). Cerza et al., in a study of 1,008,253 citizens in Rome with 13,104 PD cases, found no association between PM exposure (PM10, PM2.5, PM2.5 absorbance, and PM2.5–10) and PD incidence, using a land use regression model for exposure calculation [24]. Rumrich et al. investigated 22,189 incident PD cases in a Finnish register‐based study, matching cases with up to seven controls, and observed inverse, albeit clinically insignificant, associations between long‐term exposure (6–16 years before diagnosis) to certain PM components (sea salt, black carbon, secondary organic aerosols, and sulfate) and development of PD. The relatively low average PM exposures in Finland might explain the lack of significant findings [30]. In a newly published analysis by Gialluisi et al., PM10 contact was substantially linked to an increased risk of PD in a prospective cohort of 23,841 Italian people (213 incident PD cases over 11.2 years). An 18% increased risk was associated with every 1 μg/m^3^ rise in PM10, while the association with the second principal component, including O3/CO/SO2, and the third one, including NOx/BTX hydrocarbons, was less evident [17]. A Taiwanese study using 1060 newly diagnosed PD cases and 4240 controls, matched for age and sex, also suggested a comorbidity effect, where PM10 exposure coupled with conditions like dementia, stroke, depression, head injury, sleep disorder, and hypertension increased development of PD [34].

**TABLE 1:** Epidemiological studies on air pollution and Parkinson’s disease incidence and prognosis.

Incidence studies	Setting/population	Sample size	Parkinson’s disease case definition	Exposure assessment	Assessed confounding factors	Exposure metric	Key estimate (pollutant, increment)	Main limitations
Gialluisi et al. [17]	Italy, population‐based cohort, general population of the Molise Region, ages ≥ 35 years	23,841 participants (213 incident PD cases)	Electronic health records linkage (drug registry N04XX, ICD‐9332.0 hospital data), neurologist validation, 100% confirmation in subsample	Ordinary Kriging of 14‐station (2006–2018) air pollution data (PM10, NOx, NO, NO_2_, O_3_, CO, SO_2_, BTX) linked to residential geolocation	Age, sex, education, occupational toxic exposure, working class, smoking status, urban living (plus drinking, Mediterranean diet, physical activity, BMI sensitivity)	Principal component PC1 (tagging PM10); also directly tested average PM10	HR = 1.04 per PC1 unit; +18% PD risk per 1 μg/m^3^ PM10; PM10 > 11.65 μg/m^3^ associated with ∼14% higher PD risk	Limitations are the absence of satellite and PM_2.5_ data, possible PD misclassification, lack of pesticide contact data, and possible residual confounding
Krzyzanowski et al. [18]	U.S. Medicare beneficiaries (2000–2016)	∼9.8 million	ICD‐9 and ICD‐10 codes from Medicare claims	Predicted PM_2.5_ from spatiotemporal models linked to ZIP codes	Age, sex, race, Medicaid eligibility (SES proxy), *indicator of neurologist density, healthcare utilization*	*annual average* PM_2.5_	56% greater relative risk at median PM_2.5_; 4.2% higher risk per 1 μg/m^3^	Ecological exposure assignment; residual confounding by SES, smoking
Ritz et al. [19]	Denmark, case–control (1996–2009)	1696 PD cases, 1800 controls	Danish hospital registries (primary or secondary diagnosis)	Dispersion‐modeled NO2 at residential addresses (1971 to diagnosis)	Sex, birth year, and potential confounders (tobacco, education, occupation)	NO2 (IQR = 2.97 μg/m^3^)	9% higher PD risk per IQR increase (2.97 μg/m^3^) in modeled NO2	Exposure modeled at residential address only; no personal monitoring
Liu et al. [20]	U.S. nested case–control	1556 PD cases, 3313 controls	Self‐reported, physician‐diagnosed PD	National fine‐scale geostatistical model (home addresses, 1995–1996)	Matching factors (age, sex, race) plus lifestyle/behavioral factors	PM_2.5_, PM10, NO2	No overall association; higher risk in women (PM10) and never‐smokers (PM_2.5_)	Self‐reported PD diagnosis; exposure from spatiotemporal models
Liu et al. [21]	U.K. Biobank, prospective cohort, ≥ 60 years, mean follow‐up 9.17 years	> 210,000 participants	hospital records or self‐report with validation	Long‐term residential exposure to NO_2_ and PM_10_	Age, sex, smoking status, family history of PD	Per IQR increase in NO_2_ and PM_10_	NO_2_ HR 1.05 (1.01–1.09), PM_10_ HR 1.08 (1.03–1.13); men only: NO_2_ 1.07 (1.02–1.13), PM_10_ 1.07 (1.02–1.14); women: no significant associations	≥ 60 year only; no adjustment for BMI, occupation, SES; residual confounding possible
Shin et al. [22]	Ontario, Canada, cohort (2001–2013)	∼2.2 million adults (38,745 PD cases)	*Ontario Health Insurance Plan (OHIP) records*	Satellite‐based estimates (PM_2.5_), LUR models (NO2), interpolated methods (O3)	Individual‐ and area‐level characteristics (age, sex, SES, access to care)	PM_2.5_, NO2, O3	4% increase per 3.8 μg/m^3^ increment in PM_2.5_; positive associations for NO2 and O3	Residual confounding by access to neurological care
Chen et al. [23]	Taiwan, nationwide population‐based cohort (NHIRD), ages ≥ 50 years, followed 2005–2017	454,583 participants (18,862 incident PD cases)	Two or more outpatient visits or hospitalization with PD diagnosis from 2005 to 2017	BME spatiotemporal kriging using 83 EPA stations with monthly averages linked to residence and occupation	Age, sex, occupation, hypertension, diabetes, hyperlipidemia, COPD, lung cancer, CCI score, temperature, relative humidity, SO_2_, NO_2_	PM_2.5_ (IQR = 10.17 μg/m^3^)	HR = 1.22 (1.20–1.23) per IQR PM_2.5_; males 1.21; females 1.01	No lag analysis, missing early PM_2.5_ and lifetime/commuting exposure data, potential underdiagnosis, and unreported model performance metrics
Cerza et al. [24]	Rome, Italy, cohort	1,008,253 citizens (13,104 PD cases)	Drug prescriptions + hospital discharges	LUR models for PM/NOx; chemical dispersion model for summer O3	Individual and contextual variables (age, sex, SES, comorbidities)	PM10, PM_2.5_, NOx, O3	No association for PM or NOx; positive for O3	Exposure from LUR; possible misclassification
Jo et al. [25]	South Korea, nationwide cohort	∼N/A (national sample)	National Health Insurance Service (NHIS) database (ICD‐10 codes)	25 monitoring stations; annual avg. linked to residence	Age, sex, insurance type, comorbidities, lifestyle (smoking, alcohol, exercise)	NO2, PM_2.5_, PM10, O3, SO2, CO	NO2 associated with higher PD risk; no significant associations for others	Administrative database; no individual confounders (e.g., smoking)
Huang et al. [26]	U.K. Biobank, Mendelian randomized	312,009 with genotyping data	Self‐reported or linked health records (validated)	LUR models for annual mean pollutant levels	Age, sex, Townsend deprivation index, lifestyle, genetic variants	NO2, PM10	Positive association, modified by genetic susceptibility	Exposure at baseline only; possible selection bias
Nicole [27]	U.S. nationwide, NIEHS Sister Study (women only, originally for breast cancer), followed 2009–2018	47,108 women (163 incident PD cases)	Physician‐diagnosed PD	2‐week average pollutant concentrations at address (2003–2018), averaged to annual exposure; accounted for residential moves	Demographics (age, race/ethnicity, education), lifestyle (BMI, smoking, physical activity), census region, area type	NO_2_, PM_2.5_	NO_2_ had a positive dose–response; PM_2.5_ had no overall impact except in the Midwest; stronger relation in high‐deprivation areas and among non‐Hispanic, non‐White women	Short exposure window, potential exposure misclassification in movers, and possible underascertainment of PD cases biasing results toward the null
Yi et al. [28]	European ancestry (GWAS data: MRC‐IEU for NO_2_; International PD Genomics Consortium for PD)‐MR	Exposure: 456,380; Outcome: 33,674 cases, 449,056 controls	Physician‐diagnosed PD	5 SNPs as instrumental variables for NO_2_ exposure; F‐statistics > 10 (range 33.3–46.1)	Not applicable; no pleiotropy detected (MR‐Egger *p* = 0.684; MR‐PRESSO *p* = 0.840)	Genetically predicted NO_2_	IVW method: OR = 4.701 [1.127–19.615] per unit increase in NO_2_	Few SNPs (*n* = 5), limited power, European‐only sample, possible pleiotropy, and no individual‐level stratification
Palacios et al. [29]	U.S. male health professionals (prospective)	50,352 men	Self‐reported, confirmed by treating neurologist	Spatiotemporal model (residence‐specific PM levels)	Age, smoking, region, population density, occupation, race	PM_2.5_, PM10	No significant associations	Limited exposure variability, non‐Hispanic White male sample, and no external validation
Rumrich et al. [30]	Finland, register‐based	22,189 PD cases, matched controls	Finnish national registers (validated PD diagnosis)	Time‐weighted avg.; modeled at residence with move history (16 years prior)	Age, sex, region (matching), socioeconomic position	PM components (BC, sulfate, etc.)	No association between particulate air pollution and PD risk	Low average PM levels across Finland may contribute to lack of significant findings
Jahanshahi et al. [31]	Northern Ireland, population‐based longitudinal cohort (NILS), ages ≥ 28 years, followed 2012–2016	292,925 participants (3089 incident PD cases)	First receipt of PD‐related medication (prescription data from Enhanced Prescribing Database) after January 2012 (semester 1)	1 km annual PM_2.5_/NO_2_ models linked to biannual residential updates; 6‐month exposure with 1‐year lag	Age, sex, country of birth, education, general health, limiting long‐term illness, economic activity, religion, marital status, dependent children, cars in household, persons per room, neighborhood deprivation	PM_2.5_ (per 1 μg/m^3^; quartiles), NO2 (per 1 μg/m^3^; quartiles)	PM_2.5_ HR = 0.99 (0.96–1.02); NO_2_ HR = 0.99 (0.98–1.00); < 50 s PM_2.5_ HR = 1.05 (1.01–1.11); and Q4 HR = 1.30 (1.01–1.67); no significant effects ≥ 50 or by sex	Prescription‐based PD misclassification, outdoor‐only modeled exposure without speciation, short‐term exposure window, and possible diagnostic delay bias
Cole‐Hunter et al. [32]	Denmark, nationwide administrative cohort, adults ≥ 30 years, followed 2000–2018	3,280,190 participants	First hospital contact OR first redeemed prescription for PD‐specific medication	Annual PM_2.5_, NO_2_, O_3_w, BC at baseline residence; hybrid LUR model (0.1 × 0.1 km)	Age, sex, year, education, employment status, income, population, urbanization, green space, traffic noise, cardiovascular disease, diabetes, COPD	Per IQR increase	PM_2.5_: HR = 1.05 [1.03–1.07]; NO_2_: HR = 1.03 [1.01–1.05]; BC: HR = 1.04 [1.02–1.06]; O_3_w: HR = 0.98 [0.97–1.00]	No smoking, BMI, or occupational data; residential mobility may misclassify exposure; residual confounding possible
Kwon et al. [33]	Central California, case–control	761 PD, 910 controls	Movement Disorder Society diagnostic criteria (neurologist‐confirmed)	CALINE4 model for CO; geospatial transport model for PM_2.5_ (home and workplace, 1981–2016)	Age, sex, race, education, smoking, pesticide exposure, SES	Traffic‐related PM_2.5_, CO	Positive association for traffic‐related PM_2.5_	10‐year average with 5‐year lag; exposure misclassification possible; recall bias in self‐reported data
Chen et al. [34]	Ontario, Canada, cohort	31,577 PD cases	Health administrative databases (validated algorithms)	Residential proximity to major roads (1996 postal code)	Age, sex, diabetes, brain injury, neighborhood income, access to neurologists, other pollutants	Residential proximity to major roads	No association	Binary exposure (near vs. far); no pollutant concentration
Chen et al. [35]	Taiwan, nationwide population‐based cohort, western part, ages 40–65 years, followed 2006–2018	5,113,322 individuals (20,694 incident PD cases)	Hospital discharge diagnosis or ≥ 3 outpatient visits with ICD‐9‐CM 332 or ICD‐10 G20	Hybrid Kriging/land‐use regression using 73 monitoring stations; annual township‐level means linked to updated residential address	Age, sex, degree of urbanization, insured amount (SES), diabetes, hypertension, viral hepatitis, ambient temperature, relative humidity	PM_2.5_, PM10, NO_2_, CO, SO_2_, O_3_ (per IQR increment; IQRs: PM_2.5_ = 9.5 μg/m^3^, PM10 = 20.0 μg/m^3^, NO_2_ = 7.4 ppb, CO = 0.21 ppm, SO_2_ = 1.0 ppb, O_3_ = 4.3 ppb)	PM_2.5_ HR = 2.51 (2.45–2.58); PM10 1.25 (1.18–1.33); NO_2_ 1.76 (1.70–1.83); SO_2_ 1.15 (1.13–1.18); O_3_ 1.29 (1.25–1.33); CO nonsignificant	Observational design, diagnostic‐code outcomes, no data on smoking, pesticides, occupation, diet, or genetics, mixed land‐use uncertainty, and no individual exposure duration
Finkelstein and Jerrett [36]	Toronto and Hamilton, Canada	110,000 individuals	Physician diagnosis (Ontario Health Insurance Plan)	Fixed‐site monitors for Mn (vehicle exhaust and industrial emissions)	Age, sex, SES, other pollutant exposures (adjustment details limited)	Mn	OR = 1.034 per 10 ng/m^3^ Mn in Hamilton (earlier PD diagnosis)	Ecological exposure; small number of cases in Hamilton
Wu et al. [37]	Pearl River Delta Region, China, adults aged ≥ 60	148,274 participants	Self‐reported/doctor‐diagnosed Parkinson’s disease	20‐year exposure to PM_2.5_ constituents (BC, OM, NH_4_^+^, NO_3_^-^, SO_4_^2-^); residential address‐based models	Age, sex, lifestyle factors, socioeconomic status	Quartiles of PM_2.5_ constituent concentrations	A significant positive association was observed for all PM_2.5_ constituents	Cross‐sectional design; possible recall bias, exposure misclassification, and residual confounding
Zhang et al. [38]	U.S. Medicare beneficiaries, nationwide (2000–2016)	56.5 million (≥ 65 years)	First hospitalization for LBD (includes PDD and DLB)	Zip‐code level PM_2.5_ from validated spatiotemporal models	Age, sex, race, Medicaid (SES proxy), zip code smoking prevalence	PM_2.5_ (zip‐code level)	9% increased LBD hospitalization risk per 5 μg/m^3^ PM_2.5_; risk higher for LBD than for PD alone	Zip code level exposure; first hospitalization as proxy for diagnosis

**Prognosis studies (hospitalization, mortality)**								

Kioumourtzoglou et al. [39]	Northeastern United States, elderly cohort	∼9.8 million	First hospital admission with PD as primary diagnosis (Medicare claims)	City‐wide average PM_2.5_ from monitors (averaged annually)	Prior cardiopulmonary hospitalizations, year, age, sex, race	Long‐term PM_2.5_	Faster time to first PD hospitalization in high PM_2.5_ areas	Possible misclassification of admissions; ecological exposure
Bai et al. [40]	China, nationwide case‐crossover study, 233 cities, 2013–2017	293,357 hospital admissions	Hospital admission with principal discharge diagnosis of PD	Ambient PM_2.5_, PM_2.5–10_, NO_2_ concentrations from fixed monitoring stations; short‐term exposure	Generalized propensity score; co‐pollutants in two‐pollutant models	IQR increase: PM_2.5_: 34.5 μg/m^3^; PM_2.5–10_: 20.2 μg/m^3^; NO_2_: 19.5 ppb	PM_2.5_ 1.80% (0.71–2.90), PM_2.5–10_ 2.93% (1.44–4.45), NO_2_ 4.67% (2.90–6.47); adjusted: PM_2.5–10_ and NO_2_ significant; co‐exposure 9.72% (5.75–13.85)	Ecological exposure (ambient monitoring); residual confounding possible; urban‐only sample limits generalizability
Cole‐Hunter et al. [41]	Seven European cohorts (ELAPSE)	271,720 participants (381 PD deaths)	Underlying cause of death: PD, secondary Parkinsonism, or dementia in PD	Hybrid LUR models (2010) for PM_2.5_, NO2, BC, O3, and PM_2.5_ components	Age, sex, smoking, BMI, education, SES, year	PM_2.5_, NO2, BC	Positive associations with PD mortality; linear without lower threshold	Death certificate coding; residual confounding
Lee et al. [42]	Seoul, Korea, cohort (2002–2013)	National sample	Emergency hospital admissions for PD aggravation (primary diagnosis)	25 monitoring stations; moving avg. linked to residence	Age, sex, season, SES, comorbidity (adjustment details limited)	PM_2.5_, NO2, SO2, O3, CO (8‐day moving average)	Unit increase associated with PD aggravation (not statistically significant)	Short‐term exposure only; possible outcome misclassification
Zanobetti et al. [43]	U.S. national case‐crossover	National registry	First hospitalization for PD and diabetes (Medicare claims)	City‐wide average PM_2.5_ from monitors (2‐day avg.)	Age, sex, race (stratified), prior hospitalizations for diabetes/neurological diseases	Short‐term PM_2.5_	Increased risk of PD hospitalizations	Short‐term exposure only; no individual confounders
Chen et al. [44]	California, United States (2005–2018); statewide hospital discharge data	609,213 ED visits for PD	Visit with primary or secondary diagnosis of PD	Daily estimated PM_2.5_, CO, NO_2_ concentrations at 12 km resolution (bias‐corrected CMAQ), aggregated to ZIP‐code level	Day of year (spline), federal holidays, daily temperature, specific humidity; case‐crossover design (same month, weekday, ZIP code)	PM_2.5_ (IQR = 5.18 μg/m^3^); CO (IQR = 0.43 ppm); NO_2_ (IQR = 21.32 ppb)	CO lag 0 RR = 1.007 (1.003–1.011); PM_2.5_ and NO_2_ not significant; stronger effects in males and non‐Hispanic American Indians	PD misclassification, outdoor exposure proxy, residual comorbidity confounding, and short‐term‐only exposure
Wang et al. [45]	U.S. nationwide, Medicare Fee‐for‐Service beneficiaries (2001–2016), ages ≥ 65 years	49,121,026 beneficiaries (757,567 first hospitalizations with PD)	First hospitalization with PD diagnosis– excludes first 2 years of follow‐up to minimize prevalent severe PD	Annual PM_2.5_, NO_2_, summer O_3_ from ensemble machine learning models at 1 km resolution, linked to residential ZIP code	Age, sex, race, Medicaid eligibility, U.S. region, ZIP code‐level SES (poverty, education, housing, population density, %Black, %Hispanic), county BMI, %smokers, summer temperature/humidity; plus year trends	PM_2.5_ (IQR = 3.72 μg/m^3^), NO_2_ (IQR = 13.84 ppb), O_3_ (IQR = 10.09 ppb)	Doubly robust model: PM_2.5_ IRR = 1.08 [1.07–1.10]; NO_2_ IRR = 1.07 [1.05–1.08]; O_3_ IRR = 1.03 [1.02–1.05] (per IQR increase)	Observational design, no onset vs. exacerbation distinction, residential exposure misclassification, residual confounding, and no mobility or disease duration data

*Note:* DLB = dementia with Lewy bodies, ICD‐9/ICD‐10 = International Classification of Diseases 9th/10th Revision, IQR = interquartile range, Mn = manganese, NO_2_ = nitrogen dioxide, NOx = nitrogen oxides, O_3_ = ozone, PEG = Parkinson’s environment and gene study, PM_2.5_ = particulate matter ≤ 2.5 μm, PM_10_ = particulate matter ≤ 10 μm, SES = socioeconomic status, SO_2_ = sulfur dioxide.

Abbreviations: BC = black carbon, BME = Bayesian maximum entropy, BMI = body mass index, CCI = Charlson comorbidity index, CI = confidence interval, CO = carbon monoxide, COPD = chronic obstructive pulmonary disease, ED = emergency department, EPA = Environmental Protection Agency, HR = hazard ratio, IRR = incidence rate ratio, IVW = inverse variance weighted, LBD = Lewy body dementia, LUR = land use regression, MR = Mendelian randomization, NHIRD = National Health Insurance Research Database, OR = odds ratio, PD = Parkinson’s disease, PDD = Parkinson’s disease dementia, PM = particulate matter, PRS = polygenic risk score, RR = risk ratio, SNP = single‐nucleotide polymorphism.

Krzyzanowski et al. found a 56% greater relative risk of PD at the median PM2.5 exposure level compared to the lowest and a 4.2% higher risk for each 1 μg/m^3^ growth in PM2.5 using a large Medicare dataset, noting territorial range of differences in the PM2.5‐PD association, possibly due to a ceiling effect at higher PM2.5 levels [18]. Ritz et al. investigated 1696 PD patients diagnosed between 1996 and 2009 and 1800 controls in Denmark and found a 9% higher risk of PD per interquartile range (IQR) rise (2.97 μg/m^3^) in NO2. Notably, this association was more pronounced in individuals who had spent 20 years or more in a capital city [19]. Shin et al. identified 38,745 PD cases in a cohort of ∼2.2 million adults with an age group from 55 to 85 in Ontario, Canada, and found a positive association between PM2.5 (4% increase per 3.8 μg/m^3^ increment), NO2, and O3 exposure and PD incidence, adjusting for access to neurological care, socioeconomic factors, and occupational exposures [22]. Long‐term PM2.5 exposure and the incidence of PD were found to be significantly positively correlated in a statewide cohort research conducted in Taiwan using data from approximately 454,000 participants aged ≥ 50 years followed for up to 13 years; Chen et al. reported a 22% increased risk of PD per IQR increase in PM2.5 after adjusting for SO2 and NO2, with a notably stronger association in males [23]. In central California, Kwon et al. investigated the connection between traffic‐related PM2.5 and development of PD. Their analysis of 761 PD patients and 910 controls revealed increased PD odds when participants had 10‐year average exposures to PM2.5, assessed with a 5‐year lag, after adjusting for smoking and pesticide exposure [33]. On the other hand, after controlling for confounding variables, in a large Northern Irish cohort, Jahanshahi et al. found no general correlation between medium‐term exposure to PM2.5 or NO2 with the onset of PD. However, the study found a slight positive correlation (hazard ratio of about 1.05 per 1 μg/m^3^ increase) between PM2.5 exposure and PD risk in people under 50. The authors cautioned that prescription‐based case definition might overcount PD in younger age groups due to treatment of other dopaminergic disorders [31].

Liu et al., in a nested case–control analysis of 1556 self‐reported physician‐diagnosed PD cases and 3313 controls frequency‐matched based on sex, race, and age, found no overall association between PM or NO2 exposure and the development of PD but observed higher odds among women subjected to PM10 and never‐smokers encountering PM2.5 [20]. Additionally, prospective cohort evaluation based on the U.K. Biobank suggested increased PD risk with long‐term exposure to NO2 and PM10, particularly in men [21]. In a prospective study of 50,352 men, Palacios et al. found no significant links between PM exposure and the risk of PD. This finding remained consistent regardless of participants’ smoking status or population density, after accounting for age, smoking, region, and population density [29].

Yi et al. found evidence for a potential causal association between genetically predicted NO2 exposure and PD risk. Using a two‐sample Mendelian randomization approach (OR = 4.70; 95% CI: 1.13–19.62). Sensitivity studies verified that the results were robust and showed no signs of heterogeneity, reverse causality, or pleiotropy [28]. NOx, including NO2, has also been investigated. While Cerza et al. found a negative association between NOx and PD incidence [24], other studies, including Ritz et al., reported a positive connection between NO2 exposure and PD risk [19]. Further research by Huang et al. explored the interplay between environmental exposures and genetics in PD development. With analyzing data from 312,009 participants with full genotyping, they proposed that genetic susceptibility could modify the impact of NO2 and PM10, indicating that those with both high genetic risk and high pollutant exposure faced the highest PD risk [26]. Long‐term exposure to traffic‐related NO2 has been found to be linked to an elevated risk of PD, according to a large cohort study of 47,108 women. Stronger connections were found among women in locations with higher socioeconomic disadvantage. With the exception of the Midwest, where a definite positive correlation was discovered, there was no overall connection with PM2.5. The results strengthen the evidence that air pollution from traffic increases the incidence of PD, especially in sensitive populations [27]. Furthermore, NOx was found to be positively correlated with the incidence of PD in two large Dutch cohorts (> 50,000 participants); however, after full correction, no significant relationships were found for PM2.5, PM10, ultrafine particles, or NO2 [46]. Another study reported that exposure to NO2 was associated with a higher risk of PD but found no significant associations for PM2.5, PM10, O3, SO2, or CO [25]. In a study of 148,274 adults aged ≥ 60 in the Pearl River Delta, Wu et al. discovered that 20 years of exposure to PM2.5 constituents (black carbon, OM, NH_4_^+^, NO_3_^−^, and SO_4_^2−^) correlated with increased prevalence of PD, particularly for combustion‐related components (black carbon, OM, and SO_4_^2−^) [37].

O3 exposure has shown inconsistent associations with PD. Cerza et al. detected a positive association between chronic O3 exposure and PD incidence [24]. Kirrane et al. reported a positive correlation between O3 and fine PM and PD among farmers in North Carolina, using daily predicted pollutant concentrations and geocoded addresses, but not in Iowa. These regional differences may reflect variations in O3 levels and other environmental factors [47].

In the most recent meta‐analysis, long‐term PM2.5 exposure was significantly linked to an elevated odds of PD (OR = 1.10 per 5 μg/m^3^; 95% CI: 1.03–1.17). Both long‐term NO2 exposure (OR = 1.01 per 1 ppb) and short‐term PM2.5 exposure showed a marginal relationship (OR = 1.01 per 5 μg/m^3^). For O3, SO2, and CO, no significant correlations were discovered. Researchers indicated a lack of similar studies in highly polluted regions such as South America and Africa as a considerable limitation of evidence [48].

Studies examining other pollutants like CO, methane (CH4), nonmethane hydrocarbons (NMHCs), and total hydrocarbons (THC) have generally not found significant associations with PD incidence [25, 34]. In a meta‐analysis by Hu et al., a positive connection between CO exposure and PD risk was reported, with a 65% increased risk per 1 ppm increment in CO exposure, although significant heterogeneity was observed and included studies differed in the pollutants evaluated and in duration of exposure. This study also reported a 6% increase in relative risk of PD per 10 ppb increment in NOx exposure and positive correlations between O3 and NO2 exposures (per 1 ppb increment) and PD risk [49]. The divergent findings may be attributable to variations in traffic density, the methodologies used for exposure assessment, and the specific populations studied.

Short‐term exposure to CO was linked to an increase in PD‐related visits, according to a large California study designed by Chen et al. that included over 600,000 emergency room visits for the condition between 2005 and 2018. Neither PM2.5 nor NO_2_ showed any noteworthy correlations. According to the findings, people who already have PD may experience worsening symptoms or problems as a result of sudden increases in specific air pollutants [44]. Multipollutant models revealed that long‐term exposure to PM2.5, PM10, NO2, SO2, and O3 was independently linked to an elevated risk of PD in a large Taiwanese cohort research study of over 5 million adults followed for an average of 11.2 years. The strongest correlations were found between PM2.5 and NO2. Notably, CO was not significant after adjustment for other pollutants, and O_3_ became positively associated only in the multipollutant model [35]. In a U.K. Biobank cohort, Cheng et al. discovered that increased residential greenness was linked to a lower chance of PD over a 10.8‐year period, particularly among older adults, smokers, genetically vulnerable people, and those living in polluted or socioeconomically impoverished areas. This suggests that green spaces could reduce PD risk in part by reducing exposure to air pollution [50]. Cole‐Hunter et al. found that, after controlling for sociodemographic and health variables, each IQR increase in long‐term PM2.5, NO2, and black carbon exposure was linked to a 5%, 3%, and 4% greater PD risk in a nationwide cohort of 3.28 million adults in Denmark. Warm‐season O3 did not show any correlation [32].

Being exposed to metals has also been inspected as a potential risk factor for PD. Finkelstein and Jerrett, studying 110,000 individuals in Toronto and Hamilton and examining markers of vehicle exhaust and industrial emissions, detected a cohesion amid ambient Mn exposure and earlier PD diagnosis in Hamilton, with an odds ratio of 1.034 (1.00–1.07) per 10 ng/m^3^ increase in Mn, but no association with NO2 in Toronto [36]. In a large prospective study of female nurses (1990–2008, 425 incident PD cases), Palacios et al. reported limited evidence linking airborne metals (As, Sb, Cd, Cr, Pb, Mn, Hg, and Ni) to PD risk. This weak association was particularly observed in never‐smokers and residents of counties with populations over 250,000 [51]. In a systematic study and meta‐analysis, Aravindan et al. discovered that environmental toxins, specifically occupational exposure to industrial chemicals like dyes and methylene chloride, as well as dietary contact with persistent pollutants, are strongly linked to an increased risk of PD, which suggests that lowering toxin exposure may help reduce the burden of PD [52].

In conclusion, the existing literature presents a mixed picture concerning the association between air pollutants and the development of PD. Nevertheless, a number of large‐scale investigations have reported positive associations between exposure to PM, NOx, and O3 and an elevated risk of PD, underscoring the imperative for continued research into the role of air pollution in the pathogenesis of this neurodegenerative disorder. The heterogeneous findings across epidemiologic studies likely reflect important methodological differences. Case definitions varied considerably, ranging from clinically confirmed diagnoses to administrative records, prescription‐based algorithms, and self‐reported physician diagnoses, introducing the potential for outcome misclassification. Likewise, exposure assessment differed across studies, with estimates derived from land‐use regression models, satellite‐based approaches, monitoring networks, and residential proxies, each carrying varying degrees of exposure misclassification and limited ability to capture individual mobility or occupational exposure. Although many studies adjusted for major confounders, residual confounding remains possible, particularly for smoking, occupational exposures, pesticide exposure, socioeconomic status, and access to neurological care, which were not consistently accounted for across cohorts. Differences in regional pollution mixtures, pollutant concentrations, follow‐up duration, and population characteristics may have further contributed to between‐study heterogeneity. These methodological considerations should be taken into account when interpreting the overall body of evidence and may partly explain the inconsistent associations reported for individual pollutants.

## 4. Air Pollution, PD Prognosis

Studies investigated the association between air pollution and the possibility of hospitalization in PD patients. Kioumourtzoglou et al. assessed the potential consequence of long‐term PM2.5 exposure on event time, which is referred to as the time to first admission due to PD in an aged population in the northeastern United States. The researchers tracked almost 9.8 million individuals and found strong correlations in long‐term exposure to PM2.5 in the city and PD admissions. The possible misclassifications of hospital admissions can affect the outcomes of this study, which suggests future studies on this topic [39]. Bai et al. discovered that IQR increases in PM2.5–10 and NO2 were linked to 2.93% and 4.67% higher PD admissions, respectively, in a nationwide Chinese case‐crossover study of 293,357 PD hospital admissions (2013–2017). Co‐exposure to heavy pollutant exceedances increased admissions by up to 9.72%, suggesting that coarse PM and NO2 have independent and synergistic effects [40].

Regarding the prognostic impacts of air pollution on PD, Cole‐Hunter et al. evaluated the relationship between chronic exposure to ambient pollutants in the air and PD mortality among six European countries. In this study of 271,720 cohort respondents, 381 deaths from PD during 19.7 years of observation were found. In single‐pollutant assessment, authors found positive correlations between PD mortality and PM2.5, NO2, and black carbon and a negative association with O3. The connections of NO2, PM2.5, and black carbon with PD mortality were linear without discernible lower limits. In two‐pollutant analyses, associations with PM2.5 remained strong when adjusted for black carbon or NO_2_. According to this study, long‐term PM2.5 exposure may increase the risk of PD mortality. In this study, PD mortality was established as the underlying reason for death to be either PD, subsequent Parkinsonism, or dementia in PD [41].

A prolonged exposure to air contaminants, such as PM2.5 and NO2, failed to be strongly correlated with PD mortality in a major Dutch cohort study of 10.8 million individuals, according to Peters et al.’s study. However, limitations including reliance on death certificate information might have affected the ability to detect associations [53]. Long‐term exposure to PM2.5, NO2, and O3 was found to be strongly related to increased rates of initial hospitalization for PD, according to a national survey of nearly 49 million U.S. Medicare beneficiaries. The incidence rate ratios per IQR increase in doubly robust models were 1.08 (95% CI: 1.07–1.10) for PM2.5, 1.07 (95% CI: 1.05–1.08) for NO_2_, and 1.03 (95% CI: 1.02–1.05) for O3. As a modifiable intervention to lower PD‐related morbidity among older persons, air pollution mitigation is supported by these associations, which continued even at exposure levels below current national air quality requirements [45]. Chronic exposure to a 17‐pollutant mixture (15 PM2.5 components + NO2 + O3) was found to be significantly linked to an increase in hospitalizations for PD in a study of U.S. individuals aged 40 and older across 11 states using weighted quantile sum regression. Annual PD inpatient admissions increased by 21.2% (95% CI: 19.0%–23.4%) for every decile increase in the mixture. A 6.5% increase per decile (95% CI: 0.0%–12.2%) was seen in a reduced six‐pollutant model (EC, OC, NO_2_, SO_4_, NH_4_, and O_3_). The largest weights were given by O3, Pb, copper (Cu), and calcium (Ca). These findings highlight the neurotoxic potential of air pollutant combinations, especially O3 and metals, and encourage regulation of individual hazardous components rather than the entire amount of PM2.5 [54].

## 5. Short‐Term Exposure to Air Pollution and PD Incidence and Prognosis

Researchers also studied the possible relationship between brief exposure to air pollutants and PD. Lee et al. investigated the association of short‐term exposure to PM2.5, NO, SO2, O3, and CO with PD severity in Seoul through the National Health Insurance Service‐National Sample Cohort, Korea. From 2002 to 2013, it was found that PD aggravation was specifically linked to a unit increase in the 8‐day moving average of levels. Although not significantly different, the connections were stronger in women, patients between the ages of 65 and 74, and during the cold season [42]. Short‐term contact with fine particulates raised the possibility of hospitalizations for PD, according to Zanobetti et al.’s nationwide case‐crossover research of the short‐term impact of PM2.5 on hospitalizations along with mortality in patients with neurological diseases [43].

## 6. Cellular and Molecular Mechanisms Underlying the Pathological Effects of Air Pollutants in PD

### 6.1. Gut Microbiome

Recent experimental research has established a significant interrelation between the microbiome of the gut and PD, emphasizing the potential function of microbial dysbiosis in the pathogenesis. Evidence from rodent models has demonstrated that changes in gut bacteria can result in behavioral alterations and CNS pathology, implicating the microbiome in the onset and also progression of PD [55]. Similarly, clinical evidence has consistently reported shifts in the gut microbiota, further supporting the theory that PD may be caused by microbial dysbiosis [56–58]. Moreover, based on an in vitro experimental laboratory study, gut bacteria can influence drug bioavailability and efficacy through mechanisms such as bioaccumulation, biotransformation, and biodegradation, which in turn can alter the microbial community [59]. For example, dopamine therapy has been found to have a relationship with a reduced abundance of *Faecalibacterium*, an anti‐inflammatory, short‐chain fatty acid (SCFA)‐producing bacterium [60, 61]. Other treatments, such as catechol‐O‐methyltransferase inhibitors (iCOMT), have been linked to elevated levels of Enterobacteriaceae and *Bifidobacterium* and reduced levels of Lachnospiraceae [61–67]. Similarly, levodopa‐carbidopa intestinal gel (LCIG) and deep brain stimulation (DBS) have been shown to affect the composition of the gut microbiome, with LCIG increasing Enterobacteriaceae abundance and DBS altering taxa such as *Clostridium* cluster XIVa, *Bilophila*, *Parabacteroides*, *Pseudoflavonifractor*, and *Dorea* [61, 67, 68].

#### 6.1.1. The Gut Microbiome, α‐Synuclein, and PD Pathogenesis

Mounting evidence suggests that the collection of α‐synuclein in the gastrointestinal (GI) tract may precede its deposition in the brain, pointing to the gut as a possible site of origin for PD. α‐Synuclein, a 140‐amino acid protein expressed by the SNCA genome, is mostly located in the presynaptic terminals of neurons but is also expressed in other tissues, including the GI tract [69, 70]. Excessive expression of α‐synuclein in neurons has been shown to inhibit neurotransmitter release and disrupt cellular homeostasis [67, 71]. In PD, α‐synuclein undergoes a conformational shift into a pathological β‐sheet structure, forming cytotoxic aggregates that contribute to neurodegeneration [72–74]. Importantly, constipation and other GI symptoms, linked to accumulation of α‐synuclein, local inflammation, enhanced intestinal permeability, and oxidative stress in the enteric nervous system (ENS), frequently occur years before motor symptoms appear [57, 75–79].

Zhang et al. presented emerging evidence emphasizing that chronic exposure to fine PM2.5 may play a direct role in the pathogenesis of α‐synuclein–related neurodegenerative diseases, mainly Lewy body dementia (LBD) and PD dementia (PDD). Recent surveys have established that PM2.5 exposure helps α‐synuclein aggregation and neurodegeneration, recapitulating cardinal features of LBD, including phosphorylated α‐synuclein accumulation, neuronal loss, brain atrophy, and cognitive decline, using transgenic and humanized α‐synuclein mouse models. Additionally, depletion of α‐synuclein has been shown to diminish PM2.5‐related neurodegenerative and cognitive impacts, highlighting α‐synuclein’s pivotal mediating role. Nevertheless, the slight behavioral division between wild‐type and α‐synuclein knockout (Snca‐KO) models proposes that a longer observation duration may be compulsory to capture progressive PM‐induced impairments [38].

Experimental studies further reinforce the gut microbiota role in α‐synuclein pathology. Sampson et al. demonstrated that transfer of intestinal microbiota obtained from PD patients into mice overexpressing α‐synuclein exacerbated motor impairments, while antibiotic‐induced depletion of the microbiome alleviated these symptoms. Recolonization of the gut microbiota reversed these benefits, indicating that PD pathology may be mediated, in part, by microbiota‐dependent pathways [55]. Additionally, α‐synuclein has been established to induce the production of proinflammatory cytokines and modulate immune responses within the GI tract, potentially triggering an aberrant immune reaction that may play a role in disease progression [67, 80, 81]. Consistent with this hypothesis, phosphorylated α‐synuclein has been detected in the vagus nerve, sympathetic ganglia, and GI tract of PD patients, sometimes years before clinical diagnosis [82, 83].

#### 6.1.2. The Effects of Air Pollution on Gut Microbiome

The human GI tract is highly susceptible to exposure from fine PM pollutants through multiple pathways, including ingestion and inhalation. Mucociliary clearance mechanisms in the respiratory tract rapidly transport inhaled PM from the pulmonary compartment to the GI tract based on a human experimental study [84]. Fouladi et al. elucidated that exposure to 24‐h O3 reduced the Shannon diversity index, increased the abundance of *Bacteroides caecimuris*, and altered metabolic pathways, including those involved in L‐ornithine and pantothenate biosynthesis. Similarly, being exposed to NO_2_ was associated with a diminution in microbial taxa and an increased abundance of Firmicutes. Notably, O3 exposure accounted for approximately 11.2% of the variation in gut bacterial configuration, a substantial effect size compared to other environmental and lifestyle factors [85]. Being exposed to traffic‐related air pollution has also shown a relation to specific microbial changes, including decreased Bacteroidaceae and increased Coriobacteriaceae [86]. Based on human analysis, PM2.5 short‐term exposure has induced shifts in both beneficial and potentially harmful bacterial taxa, suggesting that air pollution dynamically affects the microbiome of the gut, potentially contributing to metabolic and inflammatory disorders [87, 88].

#### 6.1.3. Core Gut Microbial Alterations in PD

A growing body of evidence designates that PD is connected with modifications in gut microbial composition. In a study from Northeast China, Li et al. detected reduced microbial variety, species richness, and β‐diversity in PD patients, with augmented *Akkermansia* and decreased *Lactobacillus* [57]. Prominently, Boertien et al. demonstrated that dysbiosis is already present in treatment‐naïve, newly diagnosed individuals, demonstrating that these microbial shifts are not simply a consequence of dopaminergic therapy; nonetheless, the alterations seem less pronounced in early disease and may become more widespread as PD advances [89]. Across numerous studies, PD patients reliably show depletion of SCFA‐producing bacteria, including *Blautia*, *Faecalibacterium*, *Roseburia*, and *Ruminococcus*, along with enrichment of *Bifidobacterium*, *Hungatella*, *Lactobacillus*, *Methanobrevibacter*, and *Porphyromonas*. SCFAs are necessary to preserve the integrity of the gut barrier and brain–gut neurotransmitter signaling, and their depletion may have far‐reaching repercussions for systemic health, including neuroinflammatory processes [90, 91]. Thus, the gut microbiota serves as an essential mediator in the interaction between environmental pollutants and host physiology, and its disruption may play a pivotal role in the toxicological impacts of air pollution. Evidence in this regard is limited by substantial heterogeneity in study populations, geographic factors, sequencing methods, and bioinformatic pipelines, which may influence the consistency of the findings. Important confounding factors such as diet, constipation, medication use, and antibiotic exposure were not consistently controlled across studies. Furthermore, the predominance of cross‐sectional designs and reliance on 16S rRNA sequencing limited causal interpretation and detailed functional characterization of the gut microbiome changes associated with PD [92, 93]. Even though original reports on *Prevotella* were varying, subsequent work elucidated that *Prevotella copri* is diminished in PD while certain other *Prevotella* species with pathogenic potential are overrepresented [62, 66, 94]. Elevated loads of *Alistipes* and other taxa within Firmicutes and Bacteroidetes have also been noted [95].

Cluster analyses have further advanced our understanding of PD‐associated dysbiosis. Wallen et al. identified three co‐occurring bacterial clusters: enriched opportunistic pathogens (*Porphyromonas*, *Prevotella*, and *Corynebacterium*); depleted anti‐inflammatory SCFA producers (*Blautia*, *Faecalibacterium*, *Roseburia*, Lachnospiraceae, and Ruminococcaceae); and elevated carbohydrate‐metabolizing probiotics (*Lactobacillus* and *Bifidobacterium*) [96]. Genetic variants that influence α‐synuclein expression have been offered as a potential driver of these compositional alternations [97]. Meta‐analyses have verified these patterns. A systematic review by Heravi et al. reported higher abundances of *Akkermansia*, Verrucomicrobiaceae, Lachnospiraceae, and Ruminococcaceae in PD, whereas healthy controls had more *Blautia*, *Coprococcus*, Prevotellaceae, and *Roseburia* [98]. Likewise, Romano et al. confirmed a consistent decrease in SCFA‐producing taxa coupled with increased *Lactobacillus*, *Akkermansia*, and *Bifidobacterium*, although the inferred association between microbiome alterations and intestinal inflammation was mainly indirect and based on microbial composition patterns rather than direct inflammatory biomarkers or mechanistic experiments [99]. Jointly, these microbial conflicts are thought to foster a proinflammatory intestinal environment, possibly deteriorating common GI symptoms such as constipation [99]. Overall, the evidence powerfully signifies gut dysbiosis in PD pathogenesis and recommends that microbiota‐targeted interventions could demonstrate a promising therapeutic avenue [100].

**FIGURE 1:**
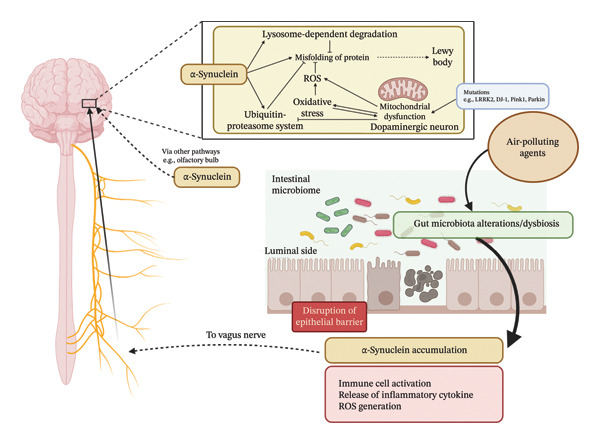
The potential gut–brain axis role in the pathogenesis of PD. α‐Synuclein misfolding and aggregation within dopaminergic neurons, leading to oxidative stress, mitochondrial dysfunction, and ultimately the generation of Lewy bodies, are central to PD pathology. Genetic mutations (e.g., in LRRK2, DJ‐1, Pink1, and Parkin) can contribute to these processes. Air‐polluting agents can induce variations in the gut microbiota (dysbiosis), potentially disrupting the intestinal epithelial barrier. This disruption may lead to α‐synuclein accumulation in the gut, triggering immune cell activation, the dissemination of inflammatory cytokines, and the creation of reactive oxygen species (ROS). Furthermore, α‐synuclein can potentially translocate from the gut to the brain through the vagus nerve and other pathways (e.g., the olfactory bulb), promoting the progression of PD.

Despite these insights, the physiological consequences of pollutant‐induced microbiome alterations remain underexplored. However, it is plausible that changes in microbial composition and function contribute to systemic toxicological outcomes, including inflammation, metabolic dysregulation, and impaired barrier function. As such, the gut microbiome represents a critical, yet underappreciated, factor in evaluating the health effects of environmental pollutants [101] (Figure 1).

#### 6.1.4. Mechanisms of Gut Barrier Disruption by PM

PM exposure has been implicated in increased oxidative stress and impaired epithelial barrier function in both pulmonary and intestinal systems. In vitro studies have demonstrated that PM exposure may promote dose‐dependent mitochondrial reactive oxygen species (ROS) production in alveolar epithelial cells, leading to cellular dysfunction [102, 103]. PM also downregulates tight junction proteins, increasing paracellular permeability in alveolar and colonic epithelial cells. These effects disrupt the epithelial barrier, allowing harmful substances to infiltrate and trigger local and systemic inflammatory responses [104]. Animal studies have confirmed these findings. In vivo experiments in mice exposed to PM showed augmented gut permeability and disrupted tight junctions in the colonic epithelium, mediated through decreased expression of key tight junction proteins [104]. Additionally, PM exposure stimulated mitochondrial ROS production and apoptosis in colonic epithelial cells, further compromising the gut barrier. These results highlight PM’s crucial function in impairing epithelial integrity and promoting inflammatory signaling in the GI tract.

#### 6.1.5. Immune and Microbial Impacts of PM Exposure

Beyond barrier dysfunction, animal studies have shown that PM exposure has profound immunological and microbial consequences. Short‐term encounters with PM10 in mice have been demonstrated by Kish et al. to change immune‐related gene transcription, increase gut permeability, and increase proinflammatory cytokine release in the small intestine. In long‐term studies, both wild‐type and interleukin‐10‐deficient (IL‐10^−/−^) mice experienced heightened colon proinflammatory cytokine expression, alterations in SCFA concentrations, and significant shifts in microbial composition. Notably, IL‐10^−/−^ mice displayed more severe histological damage, suggesting that PM exacerbates pre‐existing inflammatory conditions [105]. Also, other animal studies suggest that microbial alterations included an increased abundance of Verrucomicrobia and a diminution in Bacteroidetes alongside an increase in Firmicutes. These changes reflect a dysbiotic microbial profile often associated with inflammation and metabolic dysfunction. Interestingly, the immune response to PM exposure varied over time. By Day 14, initial increases in cytokine secretion and immune gene expression observed at Day 7 had diminished, suggesting an adaptive or suppressive immune response to continuous exposure. This effect was comparable to hyporesponsiveness observed in diesel‐exhaust‐particle‐treated human peripheral blood monocytes, which has been linked to impaired microbial clearance and increased susceptibility to infections [106].

### 6.2. Air Pollution, Neurodegeneration, and Proteinopathies

Air pollution not only affects the gut but also has significant implications for neurodegenerative diseases, including PD. A hallmark of these conditions is the aggregation of misfolded protein accumulations, such as amyloid‐β with α‐synuclein, that may be exacerbated by PM exposure [107]. Even in children and young adults, epidemiological and postmortem studies link such exposures to early amyloid and tau pathology, faster cognitive decline, and increased risk of PD [108]. PD in particular is characterized by α‐synuclein accumulation, with early symptoms including olfactory dysfunction, which precedes motor deficits. This olfactory impairment is linked to damage in the olfactory bulb, which is among the earliest sites of pathology of α‐synuclein in PD [109–114]. In a hypothesis study, the author suggests that inhaled air pollutants and other environmental toxins can initiate a “brain‐first” form of PD via the olfactory system. Drawing on data from Mexico City, detailed early α‐synuclein pathology in the olfactory bulb and brainstem of young individuals with high air pollution exposure. This points to ultrafine particles as a potential trigger for α‐synuclein misfolding [115]. Emerging data from an in vitro investigation suggest ferroptosis as a new route that goes beyond oxidative damage and inflammation. By interfering with the p53/xCT/GSH/GPX4 pathway, Theerens et al. demonstrated that urban ultrafine particles cause ferroptosis in human dopaminergic neurones, indicating a possible mechanistic link between airborne pollutants and PD neurodegeneration [116]. A newly published clinical study found that exposure to nanosized PM influenced α‐synuclein propagation in the brain. While nanosized PM alone had a limited effect, its combination with preformed α‐synuclein fibrils significantly reduced glutamate receptor 1 (GRIA1) mRNA levels in the olfactory bulb and cortex, indicating a synergistic effect on neurodegenerative processes [109]. Furthermore, Calderón‐Garcidueñas et al. identified PM within neurons of the olfactory bulb and other brain regions, highlighting its potential to penetrate the BBB and induce neuroinflammation, amyloid‐β accumulation, and α‐synuclein aggregation, with these effects detectable even in children [117]. Researchers, including Lilian Calderón‐Garcidueñas et al., discovered that young adults in Mexico City, a city with high pollution levels (PM2.5 exceeding standards of the Environmental Protection Agency), exhibited MRI‐confirmed atrophy in brain areas associated with PD. These individuals also displayed mild cognitive impairment, with MoCA scores averaging 22.8 compared to 26.2. Also, they discovered that young residents of Mexico City exhibited early signs of α‐synuclein pathology in the brainstem and substantia nigra, even in infants as young as 11 months old. These individuals also showed high incidences of combined Alzheimer’s, PD, and TDP‐43 pathologies. This suggests that prolonged exposure to PM2.5 from a young age could contribute to neurodegeneration that mirrors PD pathology and could initiate early neurodegenerative protein diseases years before any clinical symptoms become apparent [118, 119].

### 6.3. Air Pollution, Inflammation, Neuroinflammation, and Oxidative Stress in PD

Air pollutants, particularly PM2.5 and O3, affect the CNS over either direct or indirect mechanisms. Indirectly, pollutants inhaled into the respiratory system generate reactive oxygen and nitrogen species (RONS), which travel all over the body, interrupt the BBB, and alter brain homeostasis. Pollutants can directly infiltrate the CNS via the olfactory mucosa, affecting crucial brain areas including the hippocampus, cerebral cortex, and substantia nigra [15, 120, 121]. Experimental studies on rats exposed to diesel exhaust have demonstrated significant increases in TNF‐α levels in brain regions, particularly in the midbrain, which also shows elevated α‐synuclein levels [122]. Similarly, O3 exposure leads to oxidative damage, dopaminergic neuron death, and motor impairments in animal models, further linking air pollution to PD development [123–126]. PD patients appear to have heightened sensitivity to oxidative stress, particularly from air pollutants. Studies have shown that exposure to oxidative stressors like NO, CO, and NOx uplifts inflammatory markers, such as IL‐1β and tumor necrosis factor‐α (TNF‐α), in PD patients [127, 128]. Several investigations have linked PM, black carbon, O3, and NO_x_ to increased inflammatory biomarkers, TNF receptor 2 (TNFR2), C‐reactive protein (CRP), and IL‐6. For instance, a study of nonsmokers in Boston discovered positive connections between PM2.5 and CRP levels, and another study designated gender‐specific effects, finding that NO2 exposure increased CRP in women, while PM2.5–10 exposure was linked to IL‐6 and TNFR2 in men [129–131]. Furthermore, long‐lasting exposure to PM10 has also been associated with elevated inflammatory markers, supporting a consistent connection between air pollution and systemic inflammation [130–134]. Widespread metabolic changes were linked to exposure to PM2.5. CO and NO_2_ in an untargeted serum metabolomics investigation of 616 PD patients and 271 controls. Air pollution exposure affected amino acid pathways, especially tyrosine metabolism, which may have hampered dopamine synthesis, as well as lipid metabolism (increased proinflammatory leukotrienes and decreased anti‐inflammatory lipids) in PD patients. Inflammation, oxidative stress, mitochondrial dysfunction, and dopaminergic impairment are implicated in the pathophysiology of the findings, which show that PD patients have increased metabolic susceptibility to air pollution [135].

#### 6.3.1. Molecular Pathways of Oxidative Stress and Inflammation

**FIGURE 2:**
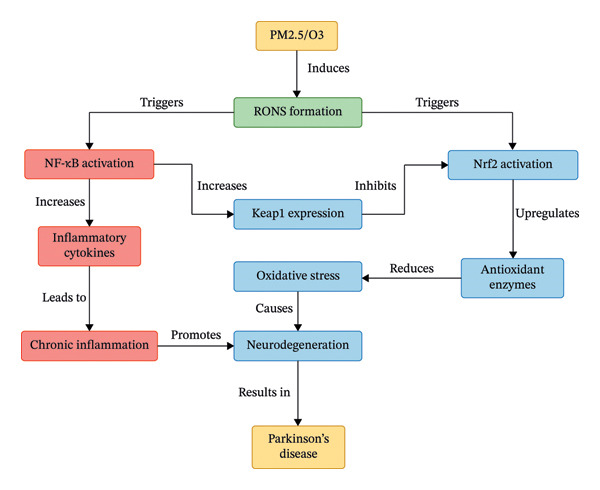
Exposure to PM2.5 and O3 triggers RONS formation, activating Nrf2, NF‐κB, TLRs, and MAPKs, leading to oxidative stress and inflammation, with Nrf2 and NF‐κB crosstalk playing a crucial role in the antioxidant and inflammatory responses. Chronic activation of these pathways can overwhelm the antioxidant defense system, contributing to neurodegeneration and tissue damage. PM2.5—particulate matter ≤ 2.5 μm.

Exposure to pollutants like PM2.5 and O3 activates molecular pathways that amplify oxidative stress and inflammation. Pollutants stimulate RONS, which activate signaling paths such as nuclear factor kappa B (NF‐κB), mitogen‐activated protein kinases (MAPKs), and Keap1–Nrf2–ARE [15, 136]. Oxidative stress triggers NF‐κB, a central regulator of inflammation, which in turn stimulates the production of inflammatory cytokines and pro‐oxidative enzymes [137–139]. Concurrently, Nrf2, a critical antioxidant transcription factor, is activated to mitigate oxidative damage but may be inactivated by prolonged pollutant exposure due to Keap1 overexpression. This inactivation reduces the expression of antioxidant enzymes like glutathione reductase and superoxide dismutase and further exacerbates oxidative stress [15, 123, 140–144]. The inconsistency between oxidative damage and antioxidant defenses underscores the vulnerability of cells to prolonged exposure to air pollutants (Figure 2).

#### 6.3.2. Neurotoxicity of Air Pollution and Glial Activation

**FIGURE 3:**
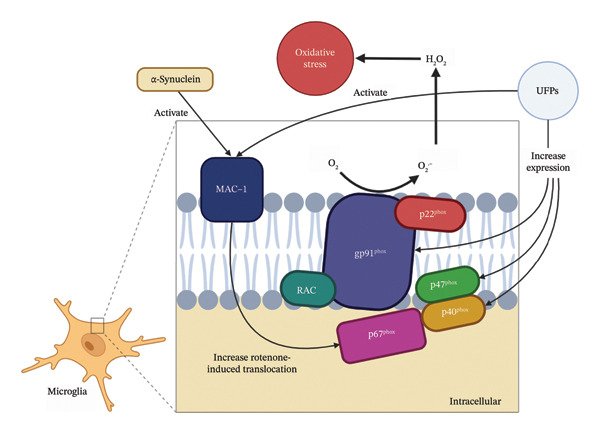
Intertwined positive feedback loops in PD: The Role of Microglial and Neuronal NOX2. This model depicts the complex interplay between microglial and neuronal NADPH oxidase (NOX2) in the context of Parkinson’s Disease, potentially exacerbated by exposure to ultrafine particles (UFPs). Initial inflammatory stimuli, such as those triggered by UFPs or other factors, activate microglial NOX2, leading to superoxide production and conversion to H_2_O_2_. This H_2_O_2_ enters neurons, elevating intracellular ROS and oxidative stress. This, in turn, upregulates neuronal NOX2 expression via ROS‐sensitive pathways (e.g., AP‐1, NF‐κB). Simultaneously, pro‐inflammatory mediators released from activated microglia contribute to neuronal damage. The resulting excessive ROS causes mitochondrial dysfunction, leading to further ROS production—a self‐perpetuating cycle within neurons. This cycle contributes to dopaminergic neurodegeneration, a hallmark of PD. The release of neuronal DAMPs further fuels microglial activation, creating a chronic inflammatory state.

The neurotoxic effects of ultrafine PM and other pollutants stem from mechanisms including excitotoxicity, oxidative stress, and inflammation. Ultrafine PM accumulates within olfactory bulb neurons, prompting the release of glutamate, glycine, and inflammatory cytokines. These neurochemical variations may drive pathological alterations perceived in polluted urban environments, such as endothelial hyperplasia and increased α‐synuclein immunoreactivity [145, 146]. PM exposure also may promote mitochondrial dysfunction, superoxide production by microglial cells, and neuronal loss, with dopaminergic neurons being particularly vulnerable due to their sensitivity to oxidative damage (Figure 3) [147–149]. PM exposure may also induce mitochondrial dysfunction, leading to permeability transition, pore opening, reduced mitochondrial potential, and reduced ATP production [148]. Chronic pollutant exposure has been shown to activate astrocytes and microglia, initially as protective mechanisms, but prolonged activation can lead to excessive RONS production, neuroinflammation, and neuronal damage. Research using human brain models has discovered that in PM2.5‐contaminated brain simulations, crossing the BBB, this pollutant may promote astrogliosis, which is related to mild neuronal loss and infiltration of microglia [150]. Further experimental findings designate that these infiltrating microglia adopt an M1 phenotype, a process driven by IL‐1β and interferon‐γ released from neurons and reactive astrocytes when exposed to PM2.5. Moreover, it has been mentioned that these M1 microglia produce additional proinflammatory mediators and nitric oxide, aggravating neuronal injury. This damage is ascertained as synaptic impairment, tau protein hyperphosphorylation, and eventual neuronal death [150].

#### 6.3.3. Epigenetic Mechanisms Linking Air Pollution to PD

In the context of PD, there is mounting evidence that environmental exposures significantly influence disease pathways using epigenetic processes, even though genetic predisposition leads to the disease risk. The hypothesis that DNA‐methylation biomarkers of cumulative contact with Pb are linked to PD is supported by clinical investigations that demonstrate that continuous Pb exposure is consistent with a unique blood DNA‐methylation signature that is more prevalent in PD patients. Mn‐exposed welders who develop parkinsonism show decreased methylation of the NOS2 gene, indicating a pollution‐induced epigenetic shift toward a proinflammatory state. Similar patterns are observed with other toxins. Exposure to pesticides, including organophosphates and organochlorines, has been linked to altered methylation patterns in PD‐related genes like MYH15, MFAP2, and KIAA0319 found in peripheral blood. While research has historically focused on pesticides and heavy metals such as Mn, the scientific community is now increasingly acknowledging that broader environmental factors, especially air pollution, may have stronger supporting evidence. Epigenomic profiling is providing a more detailed understanding of how pollutants and toxins disrupt gene expression regulation, thereby contributing to PD development, going beyond what can be determined from genomic sequencing alone [151, 152].

While mutations in genes such as SNCA, LRRK2, GBA, and MAPT confer increased vulnerability, exposure to airborne pollutants and especially fine PM2.5, NO_x_, and heavy metals can bring epigenetic modifications like DNA methylation and histone remodeling that control the expression of these PD‐related genes. Such epigenetic changes mediate the biological connection between environmental exposures and inherited susceptibility in PD, affecting neuronal survival, oxidative stress responses, and α‐synuclein aggregation. Evidence from both human and animal studies designates that air pollution exposure may potentially facilitate aberrant methylation patterns in dopaminergic neurons, by this means aggravating molecular cascades already sensitized by genetic risk variants. Even though current studies are still limited and heterogeneous, integrating epigenomic profiling with genetic analyses holds promise for revealing disease‐specific methylation signatures. These epigenetic markers could ultimately serve as biomarkers of exposure and early disease detection, expediting our perception of how air pollution fast‐tracks PD pathogenesis amongst genetically predisposed individuals [153]. In a case–control study of gene–environment interactions, it was found that long‐standing traffic‐related air pollution exposure was mainly destructive to individuals genetically susceptible to PD [154]. A substantial advancement in this context was also the identification of a PM2.5‐induced α‐synuclein strain that triggers gene expression patterns alike to those detected in LBD, supporting its potential as an experimental model linking environmental PM exposure to pathogenic α‐synuclein conformations [38].

## 7. Conclusion and Future Remarks

Studies have reported positive correlations between exposure to PM, NOx, O3, and an increased risk of PD. Beyond the incidence of PD, studies have examined the linkage between long‐term air pollution contact and the risk of hospitalization, mortality, and disease progression among PD patients, suggesting that air pollution may not only lead to the development of PD but also aggravate the clinical course and outcomes of the disorder. Several pathways have been proposed to involve the biological processes underpinning the connection between air pollution and PD. Extensive research in animal models and human studies has reported that PD is interrelated with significant modifications in gut microbial structure, including the reduction of anti‐inflammatory SCFA‐producing bacteria and the enrichment of opportunistic pathogens. Exposure to air pollution has been shown to disrupt the gut microbiome, potentially resulting in increased gut permeability, the propagation of pathogenic processes, and inflammation that may contribute to the development and progression of PD. It has been demonstrated that interaction with air pollutants can also motivate α‐synuclein to misfold and accumulation, as well as the impairment of other key proteins involved in neuronal function and homeostasis. Air pollution has been linked to oxidative stress and neuroinflammation, which can lead to the induction of multiple transcription factors. NRF2, NF‐κB, and MAPK, forcing dopaminergic neurons to malfunction and degenerate. Furthermore, the incorporation of ultrafine PM in the brain, particularly in the olfactory bulb and other vulnerable zones, has been associated with excitotoxicity, mitochondrial dysfunction, and the propagation of neuroinflammatory processes that may be responsible for the pathophysiology of PD.

Despite progress in investigating the association between air pollution and PD, findings remain inconsistent. Some studies have recognized positive links between exposure to pollutants such as PM, NOx, and O3 and an increased risk of PD, whereas other studies have not found statistically important associations. These discrepancies underscore the complexity of the interactions between environmental exposures, genetic predisposition, and any other modifiable risk factors in the pathogenesis of PD. Mechanistic studies have offered useful insights into the biological pathways linking air pollution to PD. However, the precise mediators and dose–response relationships remain unclear. Furthermore, regional differences in pollution levels, exposure assessment methods, and population characteristics may contribute to the variability in findings across epidemiological studies. There is a growing need for well‐designed, large‐scale longitudinal studies with standardized exposure assessment methods to more accurately quantify the long‐term effects of specific air pollutants on PD risk and progression. These studies should incorporate diverse populations, accounting for regional and socioeconomic differences in pollution exposure. Comprehending the interaction between genetic susceptibility and environmental exposures, such as air pollution, is critical. Future studies should explore gene–environment interactions to identify subpopulations with heightened vulnerability to pollution‐induced PD. The development of more accurate and personalized exposure assessment methods, including wearable sensors and geospatial modeling, could improve our ability to measure individual pollutant exposure and its temporal patterns. This approach would help clarify the critical windows of exposure and dose–response relationships. Experimental studies using advanced in vitro and also in vivo models should further investigate the biological pathways linking air pollution to PD. In addition, while most studies have focused on traditional air pollutants such as PM, NOx, and O3, the role of emerging pollutants, including ultrafine particles, microplastics, and nanomaterials, warrants further exploration. Beyond disease incidence, future studies should examine how persistent exposure to air pollution influences the progression and clinical outcomes of PD, such as motor and nonmotor symptom severity, hospitalization rates, and mortality. Research should also explore the effectiveness of interventions aimed at reducing air pollution exposure, particularly in high‐risk populations. Public health policies focused on improving air quality standards and urban planning could play a decisive role in attenuating the environmental burden of PD. Bridging these gaps through interdisciplinary and collaborative research will be essential for improving our understanding of the environmental factors contributing to PD and for developing effective preventive and therapeutic strategies aimed at reducing its worldwide influence.

## Author Contributions

Conceptualization: Sina Hamzehzadeh, Farshad Zare, Seyedyashar Pourebrahimian Leilabadi, Amirreza Naseri, Elnaz Gholipour‐Khalili, Sina Pakkhesal, and Mahnaz Talebi; writing–original draft: Sina Hamzehzadeh, Farshad Zare, Seyedyashar Pourebrahimian Leilabadi, Amirreza Naseri, Elnaz Gholipour‐Khalili, Sina Pakkhesal, Fatemeh Malekinejad, and Shahrzad Fakhkhari; writing–review and editing: Fatemeh Malekinejad, Shahrzad Fakhkhari, Amirreza Naseri, Mahnaz Talebi, and Sina Pakkhesal; project administration: Sina Hamzehzadeh, Farshad Zare, Seyedyashar Pourebrahimian Leilabadi, Amirreza Naseri, Elnaz Gholipour‐Khalili, Sina Pakkhesal, and Mahnaz Talebi; visualization: Sina Pakkhesal; supervision: Mahnaz Talebi and Amirreza Naseri.

## Funding

This study was financially supported by the Student Research Committee, Tabriz University of Medical Sciences (grant number: 76828).

## Disclosure

All authors approved the final version to be submitted and agreed to be accountable for all aspects of the work.

## Ethics Statement

This study was approved by the ethics committee of Tabriz University of Medical Sciences (ethic code: IR.TBZMED.VCR.REC.1404.389).

## Consent

The authors have nothing to report.

## Conflicts of Interest

The authors declare no conflicts of interest.

## Data Availability

The authors have nothing to report.
